# Immunogenicity and safety of a multi-dose quadrivalent inactivated influenza vaccine in individuals aged 6 months to 17 years: a randomized phase III trial

**DOI:** 10.1080/21645515.2019.1697595

**Published:** 2019-12-20

**Authors:** Joyce Ojeda, José Luis Arredondo, Perla Salcedo, Mercedes Paredes-Paredes, Martin Dupuy, Celine Petit, Anne Laure Chabanon, Enrique Rivas, Sanjay Gurunathan, Iris De Bruijn, Stephanie Pepin

**Affiliations:** a Global Clinical Science, Sanofi Pasteur, Mexico City, Mexico; b Instituto Nacional de Pediatría, Unidad de Investigación Clínica, Mexico City, Mexico; c Hospital General de Ecatepec “Las Américas”, Fraccionamiento las Américas, Ecatepec de Morelos, Mexico; d JM Research Clinical Research Center, Cuernavaca, Mexico; e Global Biostatistical Sciences, Sanofi Pasteur, Marcy l’Étoile, France; f Global Clinical Immunology, Sanofi Pasteur, Marcy l’Étoile, France; g Global Pharmacovigilance, Sanofi Pasteur, Campus Sanofi Lyon, Lyon, France; h Global Clinical Sciences, Sanofi Pasteur, Mexico City, Mexico; i Global Clinical Sciences, Sanofi Pasteur, Swiftwater, PA, USA; j Global Clinical Sciences, Sanofi Pasteur, Marcy l’Étoile, France

**Keywords:** Quadrivalent influenza vaccines, immunogenicity, safety, influenza, vaccination, children, adolescents

## Abstract

Annual vaccination is the most effective way to prevent seasonal influenza. Influenza vaccines in multi-dose vial (MDV) formats can facilitate timely vaccination of large populations by reducing per-dose costs and cold storage requirements compared to single-dose pre-filled syringe (PFS) formats. MDV vaccines require thiomersal or another preservative to prevent microbial contamination. We conducted a randomized, open-label trial in 302 healthy subjects aged 6 months to 17 years to evaluate the immunogenicity and safety of a quadrivalent influenza vaccine (QIV) in a thiomersal-containing MDV format compared to the licensed thiomersal-free PFS format. Subjects were randomly assigned in a 1:1 ratio to receive the MDV (n = 153) or PFS (n = 149) format. Post-vaccination hemagglutination inhibition titers for all four vaccine strains were ≥4.9-fold higher than baseline titers with no difference in magnitude between the MDV and PFS groups. Seroconversion rates per strain were also comparable between the two groups. There were no differences in reactogenicity or safety between the two vaccine formats. These results showed that the MDV format of QIV was as safe and immunogenic as the PFS format in infants, children, and adolescents. These findings support the use of MDV QIV as a resource-saving alternative for seasonal influenza vaccination.

## Main text

Influenza remains one of the world’s greatest public health challenges, accounting for up to 1 billion cases, 5 million episodes of severe illness, and 650,000 deaths worldwide every year.^1^ Vaccination against the predominant circulating influenza A and B viruses is the most effective way to prevent seasonal influenza, and annual vaccination is recommended by the World Health Organization (WHO) for risk groups including children aged 6 − 35 months, adults aged 65 years and older, pregnant women, and individuals with chronic illnesses.^2^ In recent years, most seasonal influenza vaccines have been trivalent, covering two influenza A strains (H1N1 and H3N2) and a single influenza B strain. However, since the 1980s, two genetically distinct B lineages (Victoria and Yamagata) have co-circulated globally, complicating the selection of the B lineage strain to include in vaccines ahead of each influenza season.^3^ Quadrivalent influenza vaccines (QIVs), which contain A/H1N1, A/H3N2, and a strain from each of the two co-circulating influenza B lineages, are designed to overcome this issue.^3^ Cost-effectiveness analyses suggest a switch from trivalent vaccine to QIV would help prevent excess influenza cases and deaths, and reduce burden on healthcare resources in high- and low-income settings.^4,5^ These benefits are particularly apparent in seasons where the predominant circulating B strain is of the alternate lineage to that selected for the trivalent influenza vaccine, or where strains from both B lineages co-circulate.

Since 2016, Sanofi Pasteur has produced QIV in a single-dose, preservative-free, pre-filled syringe (PFS) format (VaxigripTetra^TM^) that is now licensed in the EU and several other countries for individuals aged 6 months and older.^6^ Single-dose PFS formats of influenza vaccines are widely used and help to simplify administration, prevent dosing errors, and reduce vaccine waste.^7^ However, they also have some disadvantages – notably their cost per dose may be higher than alternative formats and they require substantial cold storage space and transport capacity.^7,8,9^ In addition, because of limitations in their filling capacity, manufacturers may not be able to meet the vaccine needs of all countries through single-dose format production, which could lead to interruptions in global supply.

Providing influenza vaccines in multi-dose vial (MDV) formats is one way to overcome many of these challenges and can facilitate vaccination of large populations within a short period of time, for example within the desired timelines of national vaccination campaigns.^9^ However, unlike most single-dose formats, MDV vaccine formats require a preservative to prevent microbial contamination after the vial has been opened. One of the most common preservatives used is thiomersal. Thiomersal has been evaluated as a safe preservative for vaccines by the WHO Global Advisory Committee on Vaccine Safety^10^ and by several other international health authorities.^11,12^

While trivalent influenza vaccines are readily available in MDV formats,^7^ QIVs are only beginning to be produced in this format.^13^ Sanofi Pasteur has developed a 10-dose MDV format of QIV, which differs from QIV in the PFS format only by the addition of thiomersal during the final blending process. Here, we report the results of an open-label, randomized phase III study designed to evaluate the immunogenicity and safety of this thiomersal-containing MDV format of QIV compared to the licensed thiomersal-free, single-dose PFS format in children and adolescents.

**Figure 1.**
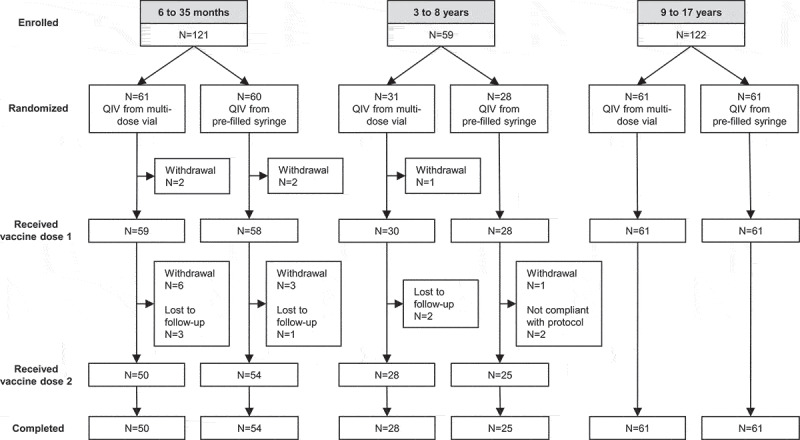
Study design and disposition of participants.

The study enrolled 302 subjects (121 subjects aged 6 − 35 months, 59 aged 3 − 8 years, and 122 aged 9 − 17 years) at three study sites in Mexico between December 19, 2017 and January 19, 2018. Subjects were randomly assigned 1:1 to receive QIV either from the MDV format (N = 153) or from the PFS format (N = 149) (Figure 1). Only about half of the planned number of subjects aged 3 − 8 years were enrolled, partly due to difficulties in enrolling unvaccinated children following extensive vaccination campaigns in elementary schools. Subjects aged 9 to 17 years received one 0.5-mL dose of QIV. Subjects aged 6 months to 8 years received two 0.5-mL doses, 28 days apart. The study was completed by 139 subjects (90.8%) vaccinated with the MDV format and 140 (94.0%) vaccinated with the PFS format. Twenty-three subjects (17 aged 6 − 35 months and six aged 3 − 8 years) did not complete the study, in most cases because they were lost to follow-up or withdrew consent. None of the subjects withdrew from the study because of an adverse event (AE). Subject demographics were similar between the recipients of each vaccine format within each age group (Supplementary Table 1).

**Figure 2.**
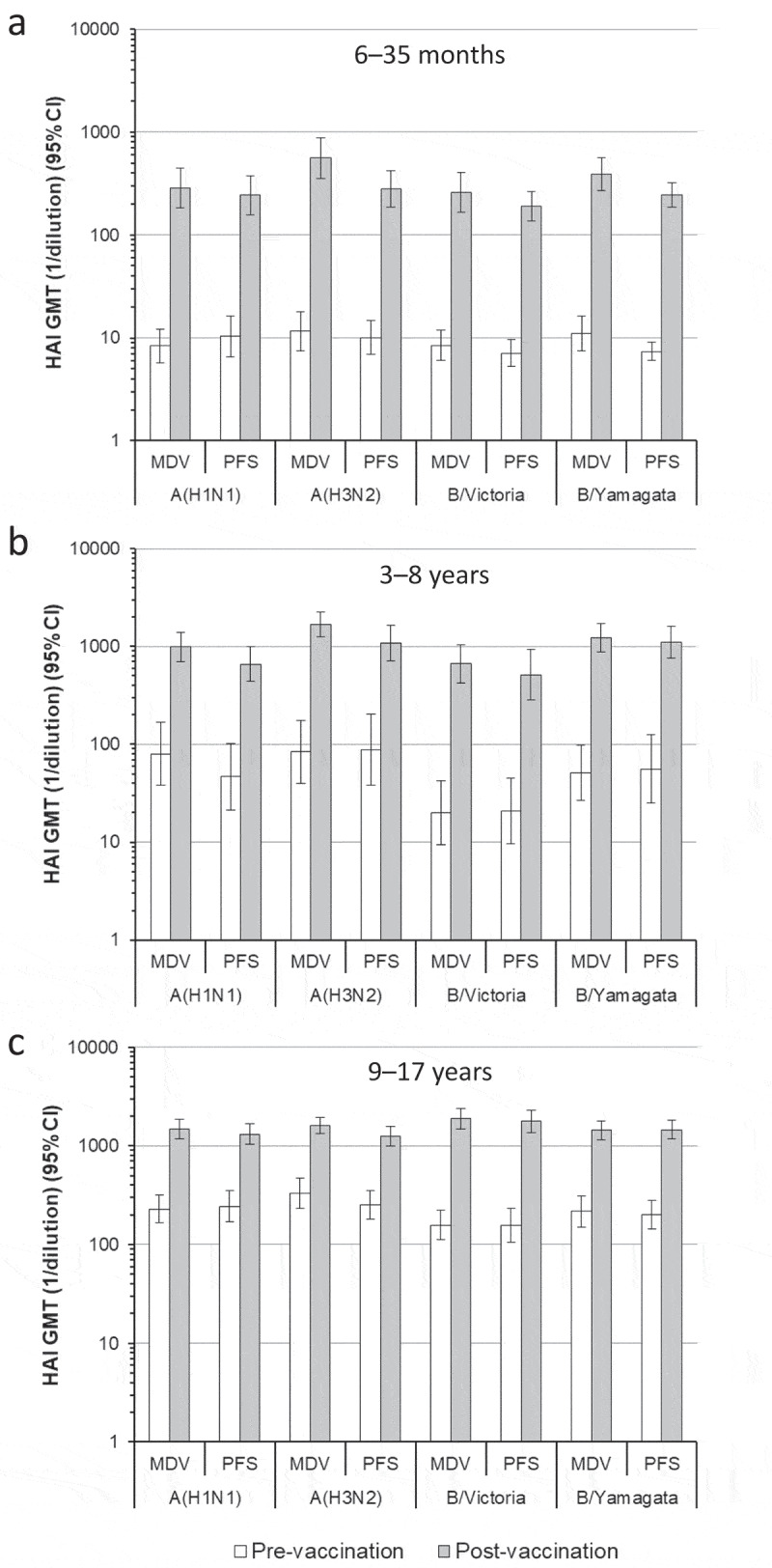
HAI antibody responses.

At baseline, hemagglutination inhibition (HAI) antibody titers for all vaccine strains were similar between the two vaccine groups within each age strata (Figure 2). In subjects aged 6 − 35 months, post-vaccination HAI antibody titers (measured 28 days after the second vaccination) were ≥24-fold higher than baseline titers in both vaccine groups for all vaccine strains (post- to pre-vaccination geometric mean titer [GMT] ratios: 31.8 − 47.3 for MDV group, 23.6 − 32.7 for PFS group), and at least 88% of MDV recipients and 87% of PFS recipients had post-vaccination titers ≥40 for each strain (Figure 2 and Supplementary Figure 1). Post-vaccination HAI titers for all strains were also higher than baseline titers in subjects aged 3 − 8 years (GMT ratios: 12.3 − 33.2 for MDV group, 12.3 − 24.6 for PFS group) and subjects aged 9 − 17 years (GMT ratios: 4.9 − 12.0 for MDV group, 5.0 − 11.4 for PFS group). Except for one 9 − 17-year-old in the PFS group, all subjects aged 3 − 17 years in both vaccine groups had post-vaccination titers ≥40 for each strain. GMT ratios were lowest among subjects aged 9 − 17 years, most likely due to higher baseline titers in this age group.

More than 85% of MDV recipients and ≥79% of PFS recipients aged 6 − 35 months seroconverted for each of the four vaccine strains (Supplementary Figure 1). Most subjects aged 3 − 8 years also seroconverted for each strain (≥79% seroconversion for the MDV group, ≥72% for the PFS group). Seroconversion rates were lowest among subjects aged 9 − 17 years (48 − 72% for individual QIV strains in the MDV group and 49 − 74% in the PFS group).

Across all age groups, post-vaccination HAI titers, GMT ratios, and seroconversion rates were similar for individual strains between subjects who received the MDV format and those who received the PFS format (Figure 2 and Supplementary Figure 1). Although post-vaccination GMTs tended to be higher in MDV recipients than in PFS recipients, the small difference is unlikely to be clinically meaningful.

Solicited injection site reactions and solicited systemic reactions were experienced by approximately half of the MDV and PFS recipients within each age group (Table 1). Pain or tenderness around the injection site was the most frequently reported solicited injection site reaction in all age groups. The most frequently reported solicited systemic reactions were irritability, abnormal crying, and malaise in subjects aged 6 − 35 months; malaise and myalgia in subjects aged 3 − 8 years; and malaise, myalgia, and headache in subjects aged 9 − 17 years. Solicited systemic reactions were reported in more infants aged 6 − 35 months in the MDV group than in the PFS group (66.0% [95% confidence interval (CI): 51.2 − 78.8%] vs. 46.3% [95% CI: 32.6 − 60.4%]); however, because the 95% CIs were overlapping, this was not thought clinically significant. Injection site reactions and systemic reactions are often experienced following influenza vaccination, and have been reported at similar frequencies in these age groups in other studies of QIV.^17,18,19^ Most solicited reactions were grade 1 (mild) in intensity and most resolved within 3 days. Overall, seven subjects experienced at least one grade 3 (severe) solicited injection site reaction (four MDV-format recipients and three PFS-format recipients) and 13 experienced at least one grade 3 solicited systemic reaction (seven MDV-format recipients and six PFS-format recipients), all of which resolved, typically within 1 − 4 days.

**Table 1.** Adverse events after vaccination.

	6 − 35 months				3 − 8 years				9 − 17 years			
	MDV format (N = 59)		PFS format (N = 58)		MDV format (N = 30)		PFS format (N = 28)		MDV format (N = 61)		PFS format (N = 61)	
Subjects experiencing at least one:	n	% (95% CI)	n	% (95% CI)	n	% (95% CI)	n	% (95% CI)	n	% (95% CI)	n	% (95% CI)
Immediate unsolicited AE	0	0.0 (0.0 − 6.1)	0	0.0 (0.0 − 6.2)	0	0.0 (0.0 − 11.6)	0	0.0 (0.0 − 12.3)	0	0.0 (0.0 − 5.9)	1	1.6 (0.0 − 8.8)
Solicited reaction	36^a^	72.0 (57.5 − 83.8)	32^b^	59.3 (45.0 − 72.4)	19^c^	67.9 (47.6 − 84.1)	19^d^	70.4 (49.8 − 86.2)	37	60.7 (47.3 − 72.9)	50	82.0 (70.0 − 90.6)
Solicited injection-site reaction	27^a^	54.0 (39.3 − 68.2)	26^b^	48.1 (34.3 − 62.2)	16^c^	57.1 (37.2 − 75.5)	16^d^	59.3 (38.8 − 77.6)	26	42.6 (30.0 − 55.9)	42	68.9 (55.7 − 80.1)
Solicited systemic reaction	33^a^	66.0 (51.2 − 78.8)	25^b^	46.3 (32.6 − 60.4)	13^c^	46.4 (27.5 − 66.1)	15^d^	55.6 (35.3 − 74.5)	30	49.2 (36.1 − 62.3)	35	57.4 (44.1 − 70.0)
Unsolicited non-serious AE	31	52.5 (39.1 − 65.7)	25	43.1 (30.2 − 56.8)	14	46.7 (28.3 − 65.7)	9	32.1 (15.9 − 52.4)	5	8.2 (2.7 − 18.1)	8	13.1 (5.8 − 24.2)
Non-serious vaccine-related	0	0.0 (0.0 − 6.1)	0	0.0 (0.0 − 6.2)	0	0.0 (0.0 − 11.6)	0	0.0 (0.0 − 12.3)	0	0.0 (0.0 − 5.9)	1	1.6 (0.0 − 8.8)
Injection site non-serious vaccine-related	0	0.0 (0.0 − 6.1)	0	0.0 (0.0 − 6.2)	0	0.0 (0.0 − 11.6)	0	0.0 (0.0 − 12.3)	0	0.0 (0.0 − 5.9)	0	0.0 (0.0 − 5.9)
Systemic non-serious vaccine-related	0	0.0 (0.0 − 6.1)	0	0.0 (0.0 − 6.2)	0	0.0 (0.0 − 11.6)	0	0.0 (0.0 − 12.3)	0	0.0 (0.0 − 5.9)	1	1.6 (0.0 − 8.8)
AE leading to study discontinuation	0	0.0 (0.0 − 6.1)	0	0.0 (0.0 − 6.2)	0	0.0 (0.0 − 11.6)	0	0.0 (0.0 − 12.3)	0	0.0 (0.0 − 5.9)	0	0.0 (0.0 − 5.9)
Serious AE	0	0.0 (0.0 − 6.1)	0	0.0 (0.0 − 6.2)	0	0.0 (0.0 − 11.6)	0	0.0 (0.0 − 12.3)	0	0.0 (0.0 − 5.9)	0	0.0 (0.0 − 5.9)

Safety was assessed according to International Conference on Harmonization guidelines^15^ in all subjects who received at least one dose of a study vaccine. Subjects recorded information about solicited reactions in a diary card for up to 7 days and about unsolicited adverse events (AEs) up to 28 days after vaccination. Any serious AEs were reported to investigators during a 6-month safety follow-up. Investigators assessed unsolicited AEs and serious AEs as unrelated or possibly related to the vaccine. Immediate unsolicited AEs were defined as those occurring within 30 min following any vaccination. AEs were coded with Medical Dictionary for Regulatory Activities (MedDRA) terminology (version 21.0). Abbreviations: CI, confidence interval; MDV, multi-dose vial; PFS, pre-filled syringe.

^a^ N = 50

^b^ N = 54

^c^ N = 28

^d^ N = 27

One subject aged 11 years in the PFS group experienced an immediate unsolicited AE (nausea), which resolved within 1 day. None of the other unsolicited AEs were considered related to a study vaccine by the investigators. No SAEs were reported and no AEs led to study discontinuation.

The results from this study showed that QIV in a thiomersal-containing MDV format had comparable immunogenicity and a similar safety profile to the licensed thiomersal-free, single-dose PFS format in infants, children, and adolescents. Although the safety follow-up period in our study was limited to 6 months, extensive evidence and reports from multiple national and international health authorities have found thiomersal to be a safe and effective vaccine preservative with no long-term safety concerns.^10,11,12,20,21^ Our results are also the first to show QIV in the MDV format is safe and immunogenic in infants aged 6 − 35 months – the youngest age QIV is licensed for, and an age group in which influenza vaccines are less often studied.^22^ This information is important given that young children are considered a risk group that is recommended annual influenza vaccination by the WHO.^2^ Moreover, MDV formats of QIV are more likely to be used for young children in low- and middle-income countries to save vaccine unit costs.^8,23^

Our study was limited in that only around half of the planned number of children aged 3 − 8 years were enrolled. Nevertheless, the overall safety of the PFS format in this age group has been previously demonstrated in a large phase III study^19^ and there was no evidence that the safety profiles of the MDV and PFS formats differed in the other age groups in our study. Additionally, our study did not evaluate the MDV format in other key risk groups for whom the vaccine is recommended, such as individuals with chronic illnesses, adults aged ≥65 years, and pregnant women; however, no safety concerns from thiomersal-containing vaccines have been identified in these populations.^9,11,12,24^

In conclusion, these results support the use of QIV in a MDV format in individuals aged 6 months to 17 years, as an alternative to the single-dose PFS format, for broad protection against influenza A and B viruses.

## Supplementary Material

Supplemental Material
