# Supplementary material for: Immunogenicity and safety of a multi-dose quadrivalent inactivated influenza vaccine in individuals aged 6 months to 17 years: a randomized phase III trial
Source: Hum Vaccin Immunother. 2019 Dec 20;16(6):1380–4. doi: 10.1080/21645515.2019.1697595 (PMC7482911; doi:10.1080/21645515.2019.1697595)
Supplement: Supplemental Material [file KHVI_A_1697595_SM3120.docx]

# Supplemental online information for “Immunogenicity and safety of a multi-dose quadrivalent inactivated influenza vaccine in individuals aged 6 months to 17 years: a randomized phase III trial”

## Study ethics

The study was approved by the ethics committees of the Instituto Nacional de Pediatria and Hispano Hospital and was conducted in accordance with the Declaration of Helsinki, the International Conference on Harmonisation Guidelines for Good Clinical Practice, and national regulations. All subjects (or their parents or legal guardians) provided written informed consent before inclusion.

## Exclusion criteria

Subjects aged 9−17 years were excluded if they had been vaccinated against influenza within 6 months before inclusion. Subjects aged 6 months to 8 years were excluded if they had received any influenza vaccine since birth or had any previous laboratory-confirmed influenza infection. Potential subjects were also excluded if they had: received any vaccine in the 2 weeks preceding study vaccination or planned to receive any vaccine in the 2 weeks following the last study vaccination; received immune globulins, blood, or blood-derived products in the preceding 3 months; known or suspected congenital or acquired immunodeficiency; received immunosuppressive therapy within the preceding 6 months; received long-term systemic corticosteroid therapy (prednisone or equivalent for >2 consecutive weeks) within the past 3 months; known hypersensitivity or history of a life-threatening reaction to the study vaccines or their components; had thrombocytopenia, a bleeding disorder, or had received anticoagulants in the 3 weeks before the study; a chronic illness that, in the opinion of the investigator, might interfere with the study assessments; or moderate or severe acute illness or infection on the day of vaccination or febrile illness (temperature ≥38.0°C). Girls were excluded if they were pregnant, lactating, or of childbearing potential and not abstinent or using contraception.

## Randomization

Subjects were randomly assigned in a 1:1 ratio by interactive response technology to receive the multi-dose vial format of the QIV or the pre-filled syringe format of the QIV. Randomization was stratified per site and per age strata (6−35 months, 3−8 years, and 9−17 years) and was performed by the permuted block method using a computer-generated randomization code supplied by the Sponsor.

## Calculation of sample size

No formal sample size calculation was performed. Each vaccine group was planned to have 180 subjects (60 subjects aged 6−35 months, 60 subjects aged 3−8 years, and 60 subjects aged 9−17 years) to ensure at least 50 subjects per age strata.

## Supplementary Figures

**
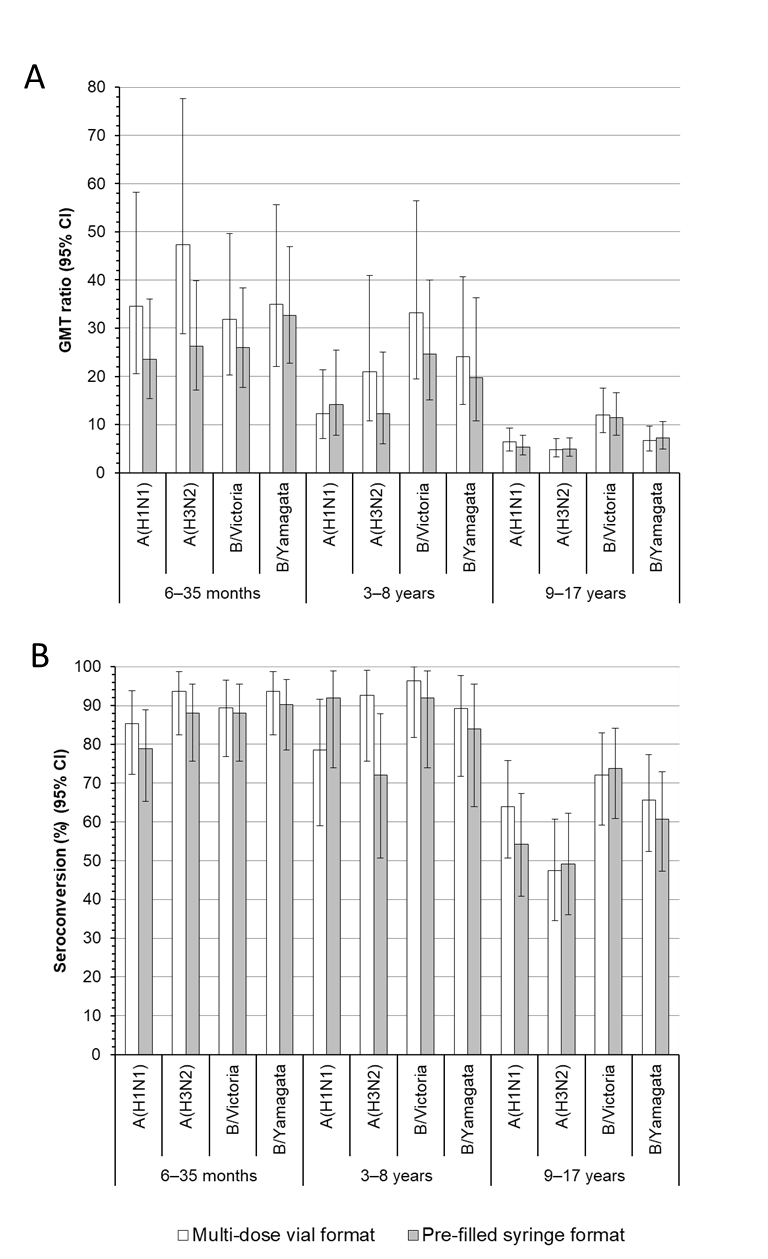
**

**Supplementary Figure 1. GMT ratios and seroconversion rates.** A. Geometric mean of the individual ratios of the post-vaccination hemagglutination inhibition (HAI) titer (28 days after the last vaccination) divided by the pre-vaccination HAI titer (day 0). B. Mean proportions of subjects with seroconversion, defined as a pre-vaccination HAI titer <10 and a post-vaccination HAI titer ≥40, or a pre-vaccination HAI titer ≥10 and a ≥4-fold increase in HAI titer. Data are from all subjects completing the study according to protocol. Abbreviations: CI, confidence interval; GMT, geometric mean titer.

## Supplementary Tables

**Supplementary Table 1. Baseline demographics of randomized subjects**

|  | **6−35 months** | |  | **3−8 years** | |  | **9−17 years** | |
| --- | --- | --- | --- | --- | --- | --- | --- | --- |
| **Characteristic** | **MDV format (N=61)** | **PFS format (N=60)** |  | **MDV format (N=31)** | **PFS format (N=28)** |  | **MDV format (N=61)** | **PFS format (N=61)** |
| Sex, n (%) |  |  |  |  |  |  |  |  |
| Male | 32 (52.5) | 33 (55.0) |  | 16 (51.6) | 18 (64.3) |  | 29 (47.5) | 34 (55.7) |
| Female | 29 (47.5) | 27 (45.0) |  | 15 (48.4) | 10 (35.7) |  | 32 (52.5) | 27 (44.3) |
| Sex ratio, male/female | 1.10 | 1.22 |  | 1.07 | 1.80 |  | 0.91 | 1.26 |
| Mean age (SD), years | 0.98 (0.90) | 0.85 (0.84) |  | 4.77 (1.73) | 4.46 (1.69) |  | 12.8 (2.46) | 12.4 (2.62) |

Abbreviation: MDV, multi-dose vial; PFS, pre-filled syringe; SD, standard deviation
